# Converting Waste into Treasure: Efficient Adsorption of Cr(VI) Using Iron-Modified Rice Straw Biochar

**DOI:** 10.3390/toxics13060458

**Published:** 2025-05-30

**Authors:** Hang Liu, Runlin Yao, Mingling Yu, Zongda Ye, Yingrui Lu, Xiaolong Yu, Jin Tang, Jianteng Sun

**Affiliations:** 1 School of Environmental Science and Engineering, Guangdong University of Petrochemical Technology, Maoming 525000, China; 2 Natural Resources Ecological Restoration Center of Guangxi Zhuang Autonomous Region, Nanning 530029, China; 3 Technical Innovation Center of Mine Geological Environmental Restoration Engineering in Southern Karst Area, Ministry of Natural Resources, Nanning 530028, China; 4 Department of Civil and Environmental Engineering, The Hong Kong Polytechnic University, Hung Hom, Kowloon 999077, Hong Kong

**Keywords:** iron-modified biochar, Cr(VI), adsorption, heavy metals

## Abstract

Hexavalent chromium (Cr(VI)) is of significant interest in the environmental field due to its high toxicity. Biochar is commonly used as an adsorbent for Cr(VI) removal from wastewater. However, its lower removal efficiency remains a persistent challenge. This study develops an iron-modified rice straw biochar through a simple precipitation and pyrolysis method and applies it for Cr(VI) removal in wastewater, which could convert waste into treasure and improve the adsorption performance of adsorbent. In the adsorption experiments, the results revealed that the adsorption efficiency of Cr(VI) reached 95.54% within 480 min (conditions: adsorbent dosage 2.67 g/L, pH 2.5, temperature 25 ± 2 °C). The Langmuir isotherm model was more suitable to describe the adsorption behavior of Cr(VI) by Fe-BC, and the fitted adsorption capacity achieved 10.03 mg/g. The experimental process was better described by the pseudo-second-order kinetic model, indicating that the adsorption process chemical adsorption was the rate-limiting step. The thermodynamic experiments revealed that the adsorption process of Cr(VI) by Fe-BC was spontaneous and endothermic. Column experiments demonstrated that a lower flow speed was beneficial to adsorption performance. Mechanistic studies highlighted the synergistic roles of electrostatic attraction, ion exchange, and reduction in Cr(VI) removal. These findings provide novel perspectives and innovative approaches for the development and application of adsorbents.

## 1. Introduction

Recently, the rapid growth of electroplating, metallurgical, and mining industries has exacerbated heavy metal pollution in aquatic environments [1,2,3]. For example, acid mine drainage with low pH and multitudinous heavy metals poses significant ecological risks for human beings [2]. The occurrence of heavy metal pollution in aquatic environments is gradually increasing and becoming a global concern. Among these contaminants, hexavalent chromium [Cr(VI)] is particularly concerning due to its high toxicity, carcinogenicity, and mutagenicity [4], which threaten human health and the environment [5,6,7].

As is well known, Cr processes two forms of trivalent chromium (Cr(III)) and hexavalent chromium (Cr(VI)) in the environment [8]. Cr(VI) is more toxic than Cr(III) [9]. For human health and environmental safety, the World Health Organization has extremely strict requirements for the maximum allowable concentration of Cr(VI) in drinking water and industrial discharge water [10]. Therefore, it is necessary to develop a suitable technology to solve the environmental problems caused by Cr(VI) in water.

Due to its adverse effects, various remediation approaches, including bioremediation, catalytic reduction, electrochemical precipitation, and adsorption, have been developed and applied to remove Cr(VI) from aquatic environments [11,12,13,14]. Among these remediation approaches, adsorption stands out for its simplicity, cost-effectiveness, and scalability [15,16,17]. Adsorbency determines whether the adsorption method can efficiently remove contaminants from water [18].

Biochar is one of the available adsorbents used to remove Cr(VI) due to its structural features (porous structure, abundant oxygen-containing functional groups, high specific surface area) [19,20]. Biochar, derived from waste biomass (e.g., rice straw, sludge), has gained attention for its porous structure and abundant functional groups [21,22,23]. However, the research results of simple biochar as a heavy metal adsorbent are not as ideal as expected. Modification of simple biochar to heighten the adsorption capacity for heavy metals has attracted significant attention [24,25]. For example, nano zero-valent iron (nZVI) and biochar composites enhanced the removal capacity of Cr(VI) [26]. However, nZVI tends to deactivate and form aggregates during practical application. To address this, iron oxide modification has been explored [27], as Fe species enhance surface hydroxyl groups and facilitate Cr(VI) reduction to less toxic Cr(III) [28]. Therefore, adding iron species (iron oxide) to biochar may be one of the most efficient ways to modify biochar.

Compared with other biomass sources, rice straw possesses the advantages of extremely abundant sources and low cost. In addition, it is reported that rice straw contains silica [29], which exhibits a unique affinity for metal ions [30]. This inherent property endows metal-modified rice straw biochar with natural advantages in related applications. Herein, iron oxide-modified biochar (Fe-BC) was prepared via precipitation and calcination and employed as an adsorbent for Cr(VI) removal. The study objectives were to: (i) synthesize Fe-modified biochar (Fe-BC) from rice straw via precipitation–calcination, (ii) evaluate its Cr(VI) adsorption performance through batch and column experiments, (iii) elucidate the underlying mechanisms using advanced characterization techniques and boost its practical application.

## 2. Materials and Experiment

### 2.1. Chemical Reagents and Materials

The chemical reagents (FeCl_3_·6H_2_O, NaOH, K_2_Cr_2_O_7_) applied in this work were of analytical grade. FeCl_3_·6H_2_O and NaOH were obtained from Aladdin reagent. K_2_Cr_2_O_7_ was purchased from Xilong Scientific Co., Ltd. (Shantou, China) All aqueous solutions were configured using deionized water. The rice straw was purchased from https://www.taobao.com/ (29 December 2023) and washed using deionized water, dried at 60 °C for one night to a constant weight, and stored at room temperature until use.

### 2.2. Preparation and Characterization of Adsorbent

Common rice straw was selected as the waste biomass. The rice straw was ground into a powder and passed through a 120-mesh sieve. The mass ratio of FeCl_3_·6H_2_O to rice straw was 1:4 (2.5 g:10 g), mixed in a 250 mL beaker (200 mL of deionized water). The mixtures were stirred for 1 h, then 1 mol/L NaOH was added to adjust the pH to 10 with stirring for another 2 h. The mixtures were washed using deionized water many times until the pH was neutral. Then, the mixtures were dried overnight to obtain the dry sample. The dry sample was calcined in a N_2_ atmosphere at 500 °C for 2 h with a heating rate of 5 °C/min, then the obtained materials were marked as Fe-BC. Unmodified biochar was also prepared using the same approach, but without the addition of FeCl_3_·6H_2_O. The unmodified biochar was marked as BC. The crystal phase structure, surface groups, BET surface aeras, BJH pore diameter, degree of graphitization, valence state of element, and Zeta potential of the obtained sample were determined using X-ray powder diffraction (XRD, XRD, Ultima IV, Rigaku, Tokyo, Japan), Fourier transform infrared spectroscopy (FTIR, TENSOR 27, Bruker, Ettlingen, Germany), N_2_ adsorption–desorption isotherm (Tristar II 3020, Micromeritics, Norcross, GA, USA), Raman spectroscopy, X-ray photoelectron spectroscopy (XPS, XPS, Thermo Fisher Scientific K-Alpha, West Palm Beach, FL, USA), and a BeNano 90Zeta analyzer (Dandong, China), respectively.

### 2.3. Batch and Column Adsorption Experiments

All batch adsorption experiments were conducted in a 200 mL conical flask, and all solutions were wrapped with aluminum foil to avoid light exposure. A total of 200 mg of adsorbent was added into 150 mL of Cr(VI) solution (initial concentration: 5 mg/L) for the adsorption experiment, then the conical flasks were placed in a magnetic stirrer with a heating function to maintain the temperature at 25 ± 2 °C. Then, the Cr(VI) sample was taken at the set time. In this work, the effects of initial Cr(VI) concentration and adsorbent dosages on the adsorption efficiency were determined, and the corresponding experimental conditions were illustrated in the corresponding legend. The adsorption isotherms, kinetics, and thermodynamic properties were also investigated. The regeneration experiments of Fe-BC utilized methods reported previously [31]. The concentration of Cr(VI) was detected with a conventional diphenylcarbazide colorimetry method (wavelength: 540 nm) using a UV spectrophotometer (Shimadzu, UV-1280, Tokyo, Japan) [32]. The adsorption efficiency (*η*, %) and adsorption capacity (*Q*, mg/g) were calculated using the following equations:

(1) $$Q\;={(C}_{0}-C_{t})\;\times \;V/m$$

(2) $$\eta \;={(C}_{0}-C_{t})/\;C_{0}\;\times \;100\%$$

Here, *C*_0_ is the initial concentration of Cr(VI) in mg/L. *C_t_* is the concentration of Cr(VI) at time *t* in mg/L. *V* is the volume of the reaction solution (L), and *m* is the weight of the adsorbent in g.

A glass chromatography column (30 cm long and 1 cm diameter) was employed for column adsorption. Firstly, the column bottom was filled with cotton (about 2 cm high) and 30 g of quartz sand. Then, 5 g of adsorbent (about 5 cm high) was added to the top of quartz sand layer. Next, another cotton layer and 30 g of quartz sand were added to the top of the adsorbent layer to prevent the adsorbent from being washed away by the current, and deionized water was added to make it fully penetrate. The quartz sand was washed using deionized water many times and dried before use. Finally, 1 mg/L Cr(VI) solution was allowed to flow into the adsorption column at a rate of 1 mL/min. The concentration of Cr(VI) from the outlet section was detected at ten-minute intervals. The flow speed was controlled using a peristaltic pump (BS100-1A).

## 3. Result and Discussion

### 3.1. Characterization

**Figure 1:**
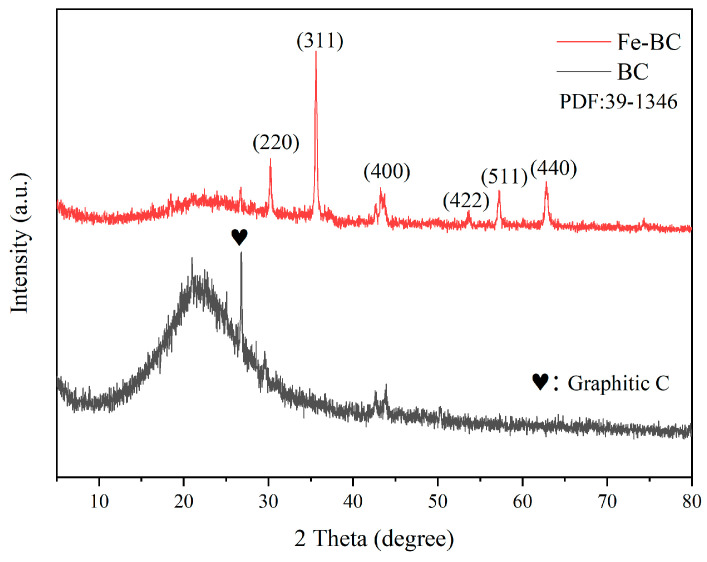
XRD patterns of the obtained BC and Fe-BC.

The crystalline structures of Fe-BC and raw BC were explored using XRD from 5–80°, and the relevant patterns are listed in Figure 1. The BC showed obvious broad diffraction peaks at approximately 21°, denoting amorphous carbon, while the diffraction peak at 26.7° was attributed to a slight graphitic carbon structure (PDF NO. 26-1079), implying that amorphous and graphitic carbon were formed under the calcine process. As for the Fe-BC, the diffraction peaks at 30.2°, 35.6°, 43.3°, 53.7°, 57.3°, and 62.9° were assigned to (220), (311), (400), (422), (511), and (440) crystalline phases of Fe_2_O_3_ (PDF NO. 39-1346), respectively. These results revealed that the Fe species was successfully loaded on the surface of the BC. In addition, the diffraction peaks of amorphous and graphitic carbon in the Fe-BC became weaker than that of BC; this phenomenon was similar to a previous study [33]. This might have been due to the successful loading of ferric oxide onto the biochar surface, which covered the diffraction peaks of amorphous and graphitic carbon.

**Figure 2:**
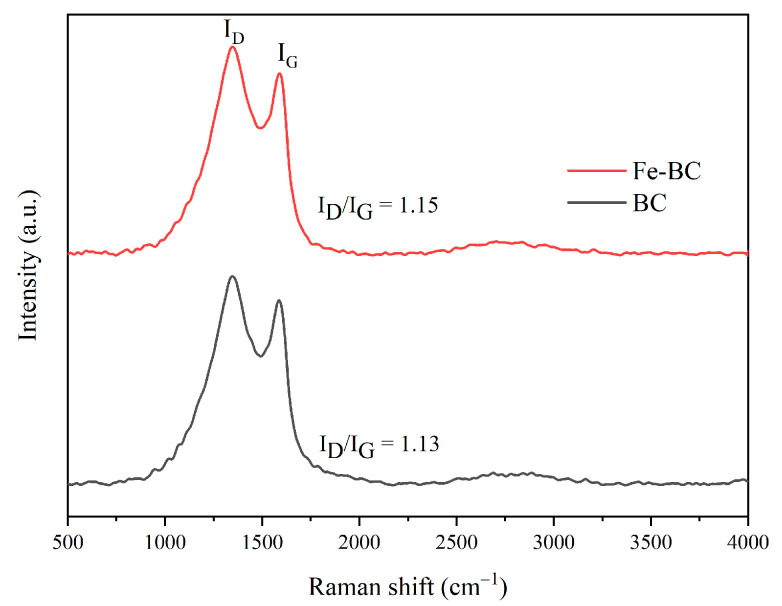
Raman spectroscopy results for BC and Fe-BC.

Raman spectroscopy was utilized to explain the surface structure defects of the BC and Fe-BC (Figure 2). Two evident characteristic peaks in the D-band (≈1350 cm^−1^) and G-band (≈1590 cm^−1^) represented amorphous and graphitic carbon, respectively. Generally, the value of I_D_/I_G_ reflects the graphitization (proportion of defects) of carbon structures, and higher I_D_/I_G_ values reveal a higher degree of structural defects in graphitic carbon. The value of I_D_/I_G_ of the Fe-BC (1.15) was slightly larger than that of the BC (1.13), demonstrating that the introduction of Fe species promoted the production of carbon defects. This might be because that the loading of metal species induced the formation of a graphitic structure under the process of metal species and carbon materials [34].

The FTIR spectrum was analyzed to explore the surface groups of BC and Fe-BC, and the results are listed in Figure S1. The FTIR spectrum revealed that BC and Fe-BC possessed broad absorbance peaks at 3250–3700 cm^−1^, which could be ascribed to the stretching vibration of -OH groups. The absorbance peaks at approximately 1390 cm^−1^ and 1090 cm^−1^ were assigned to the O-C=O groups and C-O-C groups [35], respectively. Additionally, the absorbance peak of the C=O groups was centered at approximately 1630 cm^−1^. In particular, the new absorbance peak at 570 cm^−1^ belonged to the M-O groups (Fe-O in this work) [36], indicating that the Fe species were successfully loaded onto the surface of the BC. Of course, this result was also consistent with the XRD pattern.

The pore size, pore volume, and specific surface area of the BC and Fe-BC were analyzed based on the N_2_ adsorption–desorption isotherm [37], and the results was are shown in Figure S2. The average pore diameters of the BC and Fe-BC were 7.64 and 7.26 nm based on the BJH analytical method, indicating that they possessed a mesoporous structure. Fe-BC showed a 6-fold higher surface area (108.10 m^2^/g) than BC (17.49 m^2^/g) based on the BET analytical method, enhancing active site availability. Additionally, the total pore volumes of the BC and Fe-BC were 0.040 and 0.062 cm^3^/g, respectively. The larger specific surface area provided more available active sites, which facilitated the removal of contaminants.

The XPS spectra were analyzed to investigate the chemical element and valence information of the adsorbent materials. Figure S3 presents the survey XPS spectra of the BC and Fe-BC. For the BC and Fe-BC, the C 1s and O 1s peaks were centered at approximately 285 and 532 eV, respectively. A new peak was found at 711 eV, which was ascribed to the Fe 2p [37]. This result also confirmed that the iron species were successfully loaded onto the surface of the biochar, in agreement with the results of the XRD and FTIR.

### 3.2. Adsorption Performance

**Figure 3:**
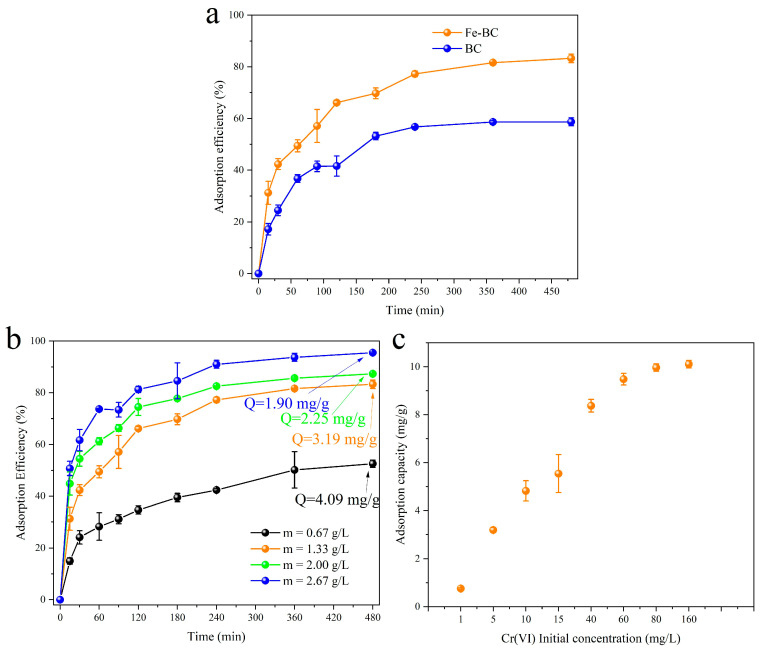
The comparison of the adsorption efficiency of Cr(VI) by Fe-BC and BC (**a**). Effect of adsorbent dosages on adsorption efficiency (**b**). Effect of Cr(VI) initial concentration on adsorption capacity (**c**). Experiment conditions: (**a**) adsorbent dosage = 1.33 g/L, Cr(VI) = 5 mg/L, V = 150 mL, pH = 2.5, and T = 25 ± 2 °C; (**b**) Cr(VI) = 5 mg/L, V = 150 mL, pH = 2.5, and T = 25 ± 2 °C; (**c**) adsorbent dosage = 1.33 g/L, V = 150 mL, t = 480 min, pH = 2.5 and T = 25 ± 2 °C.

The adsorption efficiency of Cr(VI) by BC and Fe-BC is presented in Figure 3a. The adsorption efficiency of Cr(VI) by BC was just 59.80% within 480 min. However, the adsorption efficiency of Cr(VI) by Fe-BC was 83.29% under the same conditions; an increase of nearly 24 percentage points. This indicated that the adsorption capacity of Cr(VI) by Fe-BC was indeed markedly enhanced due to the addition of iron oxide increasing the adsorption active sites. It was also demonstrated that that it is feasible to modify biochar with iron species for improving the adsorption capacity of heavy metals.

The influence of the Fe-BC dosage on the adsorption efficiency of Cr(VI) is shown in Figure 3b. The adsorption efficiency was just 52.55% when the dosage of Fe-BC was 0.67 g/L. The adsorption efficiency of Cr(VI) increased from 52.55% to 83.29% when increasing the adsorbent dosage from 0.67 g/L to 1.33 g/L within 480 min, accompanying an increase of nearly 30 percentage points. This was mainly due to the increased dosage of the adsorbent, which provided available active sites and thus facilitated the adsorption process. When the dosage of the adsorbent was further increased to 2.0 and 2.67 g/L, the adsorption efficiency of Cr(VI) was 87.31% and 95.54%, respectively. The adsorption efficiency did not exponentially increase, and this result was similar to a previous study [38]. At this point, the improvement in adsorption efficiency was not as significant as before, possibly because as the amount of adsorbent increased, although more active sites were provided, a large amount of the adsorbents may also have undergone agglomeration. The influence of the initial concentration of Cr(VI) on the adsorption capacity is shown in Figure 3c.

The adsorption capacity was also increased with the increase in Cr(VI) initial concentration. In addition, the comparisons of Fe-BC with other adsorbents reported in the existing literature [6,9,39,40] are displayed in Table S1. Overall, Fe-BC achieved high Cr(VI) removal efficiency even at a lower dosage, implying that the adsorbent in this study possessed application potential.

The effect of solution pH on adsorption efficiency is shown in Figure S4. The adsorption efficiency decreased with increases in pH. As shown in Figure S5, the pH_pzc_ (point of zero charge) of Fe-BC was approximately 3, implying that the surface of the Fe-BC was positively charged at pH < 3. At this point, the Fe-BC could adsorb Cr(VI) through electrostatic attraction. In contrast, electrostatic repulsion occurred with increases in pH, leading to a rapid decrease in adsorption efficiency.

The regeneration performance of Fe-BC was studied via adsorption–desorption experiments, as revealed in Figure S6. The adsorption efficiency gradually decreased from 83.29% to 45.57% as the Fe-BC was recycled. This might have been due to the performance of the adsorbent decreased with increasing cycles [31]. Generally, Fe-BC could still maintain a certain adsorption capacity for Cr(VI).

### 3.3. Adsorption Isotherms

In this work, Langmuir and Freundlich isotherms were used to fit the experimental data. This was valuable in studying the adsorption mechanism and the structure of the adsorption layer via the adsorption isotherms. The Langmuir and Freundlich isotherms are described by the following equations:

Langmuir isotherm:

(3) $$q_{e}=q_{m}K_{L}C_{e}/(1+K_{L}C_{e})$$

Freundlich isotherm:

(4) qe=kFCe1n

Here, *C_e_* represents the equilibrium concentration of Cr(VI) in mg/L. *q_e_* and *q_m_* represent the equilibrium adsorption capacity and predicted maximum adsorption capacity, respectively, in mg/g. *k_L_* and *k_F_* represent the Langmuir and Freundlich constants, respectively, in L/mg and (mg/g) (L/mg)^1/*n*^.

**Figure 4:**
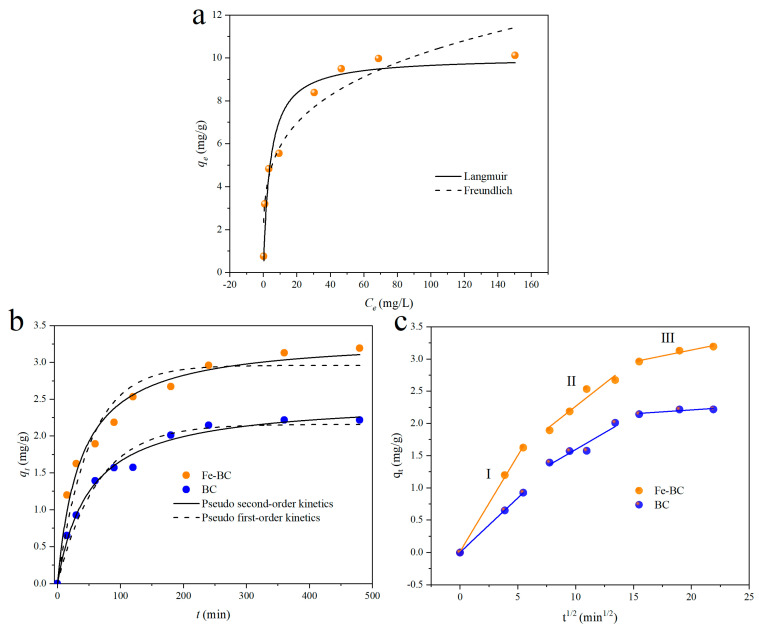
Adsorption isotherm for Cr(VI) removal by Fe-BC (**a**) and adsorption kinetics for Cr(VI) removal by BC and Fe-BC (**b**,**c**).

The fitted results and the parameters are presented in Figure 4a and Table 1. The adsorption capacity of Cr(VI) by Fe-BC increased with the initial concentration and finally achieved equilibrium. As shown in Table 1, the coefficient of determination (*R*^2^ = 0.9309) of Langmuir isotherm was higher than that of the Freundlich isotherm (*R*^2^ = 0.9181). The fitting curves of the Langmuir isotherm are closer to the experimental data, implying that the Langmuir isotherm was more suitable to describing the adsorption behavior of Cr(VI) by Fe-BC. The value of *q_m_* was 10.03 mg/g, which was close to the equilibrium adsorption capacity (*q_e_* = 10.11 mg/g). This also indicated that the adsorption process was homogeneous. In addition, the values of 1/*n* were between 0 and 1, suggesting that the adsorption process was easy [41].

### 3.4. Adsorption Kinetics

In this work, pseudo-first-order, pseudo-second-order, and intra-particle diffusion kinetic models were utilized to fit and analyze the experimental data for investigating the rate zero-controlling step and potential adsorption mechanism. The three kinetic models are represented by the following equations:

Pseudo-first-order model:

(5) $$q_{t}=q_{e}(1-e^{{-k}_{1}t})$$

Pseudo-second-order model:

(6) $$q_{t}=k_{2}q_{e}^{2}t/(1+k_{2}q_{e}t)$$

Intra-particle diffusion model:

(7) $$q_{t}=k_{p}t^{0.5}+C$$

Here, *q_t_* (mg/g) represents the adsorption capacity of the adsorbent for Cr(VI) when the reaction time is *t*. *k*_1_ (min^−1^), *k*_2_ (g/(mg·min), and *k_p_* (g/(mg·min)) represent the rate constants of the three kinetics models, respectively; *C* (mg/g) represents the intercept of the intra-particle diffusion model.

The adsorption kinetics curves are described in Figure 4b,c. In addition, the correlation coefficients (*R*^2^) and relevant kinetic parameters are summarized in Table S2. As displayed in Figure 4b and Table S2, for the original BC and Fe-BC, the pseudo-second-order kinetic model was suitable for fitting the experimental data because of its higher *R*^2^ values. Additionally, the calculated adsorption capacity (*q_e_*, _*cal*_) based on the pseudo-second-order model was slightly higher than the experimental adsorption capacity (*q_e_*_, *exp*_). Therefore, these results indicate that the adsorption process of Cr(VI) by BC or Fe-BC followed the pseudo-second-order kinetic model. As is well known, the pseudo-second-order kinetic model is based on the assumption that the rate-limiting step may be chemical adsorption. Therefore, these results demonstrate that chemical adsorption was the rate-limiting step in this work [42].

In this work, to further reveal the adsorption mechanism and behavior, intraparticle diffusion was employed. The fitting curves and rate constants are shown in Figure 4c and Table S2. It is clear that three stages existed during the adsorption process of Cr(VI). The first stage was assigned to the external surface adsorption stage, and its rate constant *k_p_*_1_ for BC and Fe-BC was 0.1685 and 0.2987 g/(mg·min), respectively, suggesting that the introduction of iron oxide to BC benefited the adsorption of Cr(VI). The second stage was the gradual adsorption stage; the values of the rate constant *k_p_*_2_ for the BC and Fe-BC were 0.1045 and 0.1409 g/(mg·min), respectively. The third stage was regarded as the final equilibrium stage. As shown in Table S2, the values of *C*_1_ in the first stage for the BC and Fe-BC were close to 0, yet the values of *C*_2_ and *C*_3_ were significantly greater than 0, suggesting that the fitting curves of the second and third stages could not pass through the origin. These results indicate that the adsorption of Cr(VI) by BC and Fe-BC was controlled by both surface diffusion and intra-particle diffusion [43]. Moreover, the rate constant *k_p_*_1_ in the first stage for BC or Fe-BC was greater than that of the rate constants *k_p_*_2_ and *k_p_*_3_, demonstrating that the adsorption rate was mainly determined by the external surface adsorption.

### 3.5. Adsorption Thermodynamics

Adsorption thermodynamic experiments were carried out to study the adsorption process of Cr(VI) by Fe-BC. The following equations were used to fit the adsorption process to obtain the thermodynamic parameters:

(8) $$\Delta G^{0}=-RTlnK_{c}$$

(9) $$K_{c}=q_{e}/C_{e}$$

(10) $$lnK_{c}={\Delta S}^{0}/R\;-\;{\text{Δ}H}^{0}/RT$$

Here, Δ*G*^0^ (kJ/mol) represents the change in Gibbs free energy. *R* represents the gas constant (8.314 J/(mol·K)), *T* represents the adsorption temperature (K), and *K_c_* represents the equilibrium constant (L/g). *q_e_* represents the adsorption capacity at equilibrium (mg/g), and *C_e_* represents the Cr(VI) equilibrium concentration (mg/L). Δ*S*^0^ (J/mol/K) and Δ*H*^0^ (kJ/mol) are the standard entropy change and enthalpy change.

**Figure 5:**
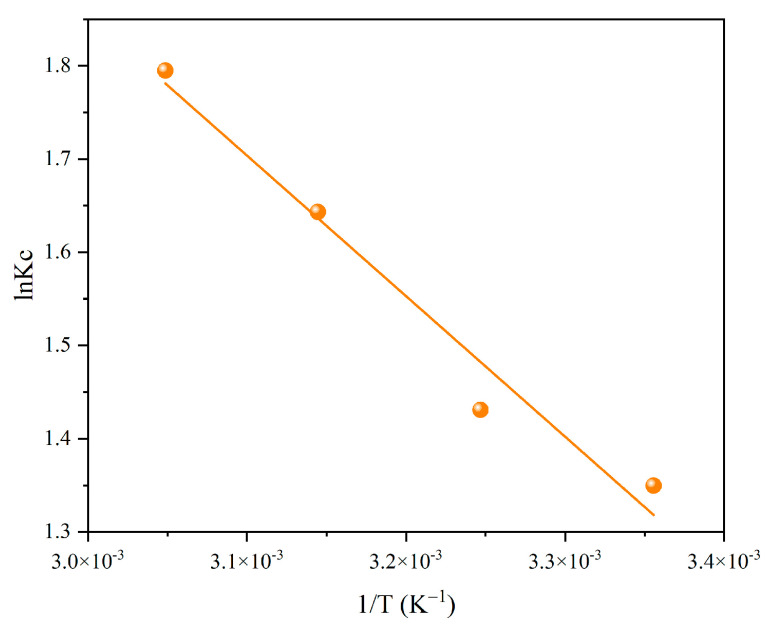
Thermodynamic curve for the adsorption of Cr(VI) by Fe-BC.

The thermodynamic curve for Cr(VI) adsorption by Fe-BC is displayed in Figure 5. The thermodynamic parameters, including Δ*G*^0^, Δ*H*^0^, and Δ*S*^0^, were calculated and are listed in Table S3. The values of Δ*G*^0^ under different temperatures were −3.27, −3.80, −4.33, and −4.86 kJ/mol, respectively. The values of Δ*G*^0^ were less than 0, implying that the adsorption process was spontaneous. Additionally, the values of Δ*G*^0^ reduced with increasing experimental temperatures, suggesting that increasing the temperature was beneficial to the spontaneous adsorption reaction. The value of Δ*H*^0^ was 12.54 kJ/mol, which was greater than 0, showing that the adsorption process was endothermic. Moreover, the value of Δ*S*^0^ was higher than 0, suggesting that the adsorption process was more likely to occur spontaneously at a higher temperature, and that Cr(VI) was firmly bound to the reactive sites of Fe-BC [19].

### 3.6. Dynamic Adsorption Experiment

**Figure 6:**
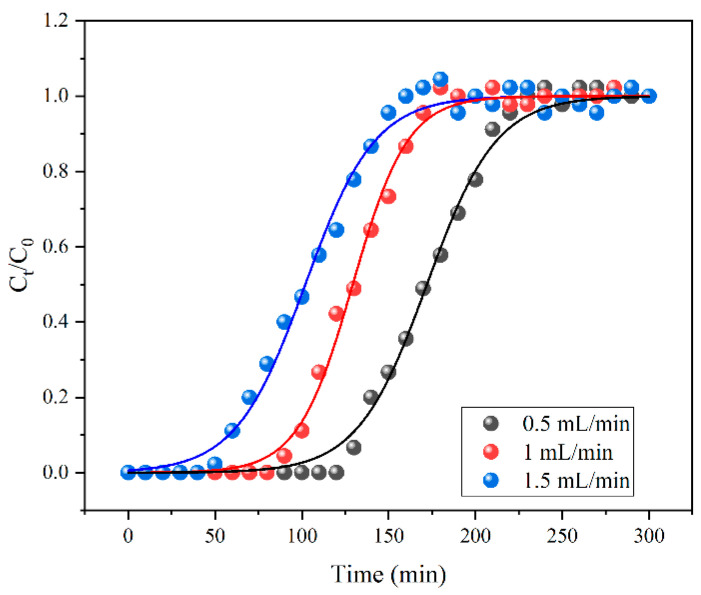
Results of the dynamic adsorption experiment.

To better explain the adsorption performance, a dynamic adsorption experiment was carried out [44]. The application potential of Fe-BC was evaluated by analyzing the breakthrough curve of the adsorbent. As displayed in Figure 6, the breakthrough time decreased when the flow speed increased from 0.5 to 1.5 mL/min. This might have been because the higher flow rate led to insufficient contact between the adsorbate and adsorbent. It was also indicated that the lower flow speed was beneficial to the adsorption process. In general, the results of this work also hinted that Fe-BC was an available and potential adsorbent for heavy metal ions.

### 3.7. Probable Adsorption Mechanism

To further research the probable adsorption mechanism of Cr(VI) removal by Fe-BC, the after reaction of Fe-BC (marked as Fe-BC-Cr) was analyzed. The structural information of the obtained Fe-BC-Cr was detected using FTIR and XPS. As revealed in Figure S1, the absorbance peak of the -OH groups was weakened after Cr(VI) elimination, indicating that the -OH groups took part in the adsorption process. In addition, compared with Fe-BC, the absorbance peak of the M-O groups (Fe-O and Cr-O) in the obtained Fe-BC-Cr was slightly enhanced, which suggested that the Cr species were successfully adsorbed onto the surface of the Fe-BC.

**Figure 7:**
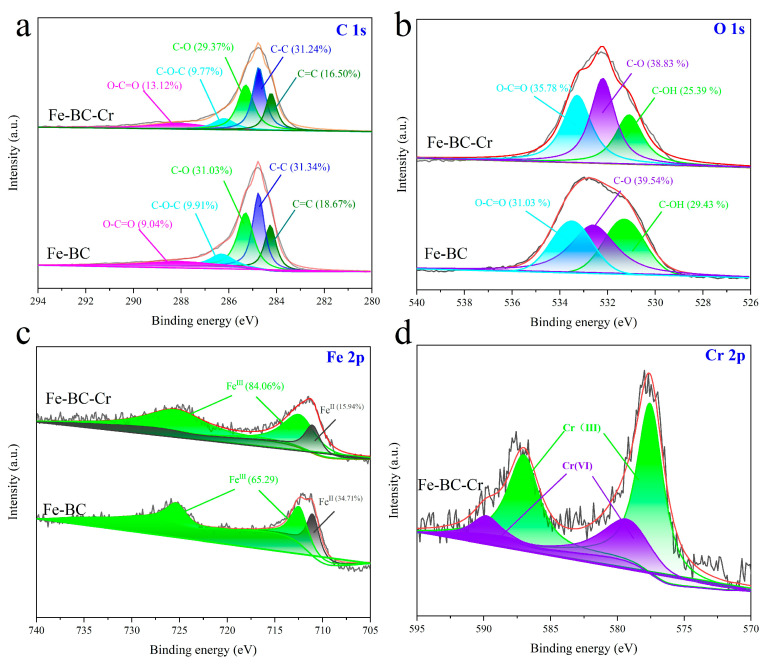
XPS spectra of Fe-BC and Fe-BC-Cr. (**a**) C 1s, (**b**) O 1s, (**c**) Fe 2p, (**d**) Cr 2p.

As shown in Figure S3, the characteristic peaks of Fe 2p, C 1s, and O 1s also appeared in the Fe-BC-Cr. Compared to the Fe-BC, a new peak centered at 575–590 eV was attributed to the Cr 2p signal, indicating that Cr was successfully adsorbed onto the surface of the Fe-BC. This result was also consistent with the FTIR spectrum. The high-resolution XPS spectra of the Cr 2p, Fe 2p, C 1s, and O 1s signals could provide more useful and accurate information for understanding the adsorption process. The C 1s was divided into C=C, C-C, C-O, C-O-C, and O-C=O bands (Figure 7a) with binding energies of 284.2, 284.7, 285.2, 263.3, and 288.1 eV [6,27], respectively. After Cr(VI) adsorption, the proportions of the C-O and C-O-C bands decreased from 31.03% and 9.91% to 29.37% and 9.77, respectively, yet the proportion of O-C=O increased from 9.04% to 13.12%. The O 1s spectra could be divided into three prominent peaks at approximatively 531.3, 532.6, and 533.5 eV (Figure 7b), attributed to C-OH, C-O, and O-C=O bands, respectively [19]. Due to the involvement of the C-O and O-C=O band in Cr(VI) removal, the proportion changed. In particular, the proportion and binding energy of C-OH were both reduced after Cr(VI) adsorption. It was reported that C-OH is an important site for Cr(VI) adsorption [19]. As for Fe 2p (Figure 7c), the proportion of Fe(II) decreased and Fe(III) increased after Cr(VI) adsorption, suggesting that the Fe species was involved in the reaction process. As shown in Figure 7d, the Cr species had trivalent and hexavalent chromium on the surface of Fe-BC-Cr, demonstrating that the Cr species underwent chemical reactions on the surface of the adsorbent [36].

In general, the adsorption process of Cr(VI) on the surface of Fe-BC could be described as follows: (1) Adsorption process: abundant surface active site, pores and functional groups could produce van der Waals forces between Cr(VI) and Fe-BC [45]. (2) Electrostatic attraction process: the surface functional groups were protonated and positively charged, promoting the negatively charged Cr(VI) adsorption (HCrO_4_^−^ was the primarily form for pH values lower than 4 [46]). (3) Ion exchange process between Cr(VI) and the surface -OH groups. (4) Reduction process: the redox reaction of Cr(VI) happened because of the Fe(II) in the Fe-BC [28].

## 4. Conclusions

Synthesizing efficient adsorbents from waste biomass offers a potential scheme for controlling heavy metals and addressing water pollution problems. Fe-modified rice straw biochar (Fe:biomass = 1:4) was prepared via precipitation and calcination. The obtained adsorbent was applied to the adsorption of Cr(VI) form wastewater. The adsorption experiment demonstrated that Fe-BC possessed a better adsorption capacity than raw rice straw biochar, reflected in the superior physicochemical properties of the Fe-BC. The adsorption efficiency of Cr(VI) by Fe-BC reached 95.54% within 480 min (conditions: adsorbent dosage, 2.67 g/L; pH, 2.5; temperature, 25 ± 2 °C). Langmuir isotherms and the pseudo-second-order kinetics model were the most suitable for describing the adsorption process. The thermodynamic experiments proved that high temperatures are more favorable for adsorption. Ion exchange, electrostatic attraction, complexation, and reduction are the main adsorption mechanisms. Generally, static and dynamic experiments demonstrated that it is feasible to use iron-modified biochar as an adsorbent to eliminate heavy metals from wastewater.

## Figures and Tables

**Table 1:** Parameters and correlation coefficients of the Langmuir and Freundlich isotherms.

Langmuir Isotherm			Freundlich Isotherm		
*q_m_* (mg/g)	*k_L_* (L/mg)	*R* ^2^	*k_F_* ((mg/g) (L/mg)^1/*n*^)	1/*n*	*R* ^2^
10.03	0.25	0.9309	3.34	0.25	0.9181

## Data Availability

The data will be made available upon request.

## Supplementary Materials

The following supporting information can be downloaded at: https://www.mdpi.com/article/10.3390/toxics13060458/s1, Figure S1. The FTIR spectrum of BC, Fe-BC and Fe-BC-Cr. Figure S2. N_2_ adsorption-desorption isotherm (a) and pore size distribution (b) for BC and Fe-BC. Figure S3. The survey XPS spectra of BC, Fe-BC and Fe-BC-Cr. Figure S4. The effect of solution pH on the adsorption efficiency. Figure S5. The Zeta potential of Fe-BC. Figure S6. Regeneration performance of Fe-BC. Table S1 The comparison of Fe-BC with other adsorbents. Table S2. The relevant parameter of adsorption kinetics for Cr(VI) removal by BC and Fe-BC. Table S3. The thermodynamic parameters of Δ*G*^0^, Δ*H*^0^ and Δ*S*^0^.
