# Supplementary material for: Converting Waste into Treasure: Efficient Adsorption of Cr(VI) Using Iron-Modified Rice Straw Biochar
Source: Toxics. 2025 May 30;13(6):458. doi: 10.3390/toxics13060458 (PMC12197260; doi:10.3390/toxics13060458)
Supplement: Supplementary file 1 [file toxics-13-00458-s001.zip › toxics-3616648-supplementary.pdf]

Supplementary information for

Converting Waste into Treasure: Efficient Adsorption of Cr(VI) Using Iron-Modified Rice Straw Biochar

Hang Liu [1,2,3], Runlin Yao [4], Mingling Yu [4], Zongda Ye [2,3], Yingrui Lu [2,3], Xiaolong Yu [1], Jin Tang [1,*] and Jianteng Sun [1,*]

[1] School of Environmental Science and Engineering, Guangdong University of Petrochemical Technology, Maoming 525000, China
[2] Natural Resources Ecological Restoration Center of Guangxi Zhuang Autonomous Region, Nanning 530029, China
[3] Technical Innovation Center of Mine Geological Environmental Restoration Engineering in Southern Karst Area, Ministry of Natural Resources, Nanning 530028, China
[4] Department of Civil and Environmental Engineering, The Hong Kong Polytechnic University, Hung Hom, Kowloon 999077, Hong Kong
* Correspondence: jintang117@foxmail.com (J.T.); sunjianteng@zju.edu.cn (J.S.)

Table S1 The comparison of Fe-BC with other adsorbents

Adsorbent	Cr(VI) concentration (mg/L)	Dosage (mg/L)	pH	Removal capacity (mg/g)	Removal efficiency (%)	Refs.
FeBC	10	8	2	—	above 99.0%	[40]
Pine needle biochar	50	10	2	4.18	~83.6%	[6]
Landfill leachate sludge biochar	50	10	2	~4.90	~92%	[35]
Magnetic biochar	100	10	3	8.35	~84.3%	[9]
Fe-BC	5	2.67	2.5	1.90	95.54%	This work

*References can be found in the main text.

Table S2. The relevant parameter of adsorption kinetics for Cr(VI) removal by BC and Fe-BC.

Adsorbent	$q_{e,exp}$ (mg/g)	Pseudo-first-order kinetic model			Pseudo-second-order kinetic model		
		$q_{e,cal}$ (mg /g)	k_1 (min^{-1})	R^2	$q_{e,cal}$ (mg /g)	k2 (g/(mg • min)	R^2
**BC**	**2.22**	**2.15**	**0.016**	**0.9695**	**2.49**	**0.0079**	**0.9879**
**Fe-BC**	**3.19**	**2.96**	**0.020**	**0.9337**	**3.34**	**0.0081**	**0.9792**

Adsorbent	Film diffusion			Intra-particle diffusion			Intra-particle diffusion		
	k_{p1} (g/(mg • min))	C_1 (mg /g)	R^2	k_{p2} (g/(mg • min))	C_2 (mg /g)	R^2	k_{p3} (g/(mg • min))	C_3 (mg /g)	R^2
**BC**	**0.1685**	**-0.0006**	**0.9999**	**0.1045**	**0.5492**	**0.8594**	**0.0119**	**1.9702**	**0.6185**
**Fe-BC**	**0.2987**	**0.0093**	**0.9977**	**0.1409**	**0.8559**	**0.8921**	**0.0365**	**2.4089**	**0.9141**

Table S3. The thermodynamic parameters of ΔG^0, ΔH^0 and ΔS^0.

T/K	lnKc	ΔG^0 (kJ/mol)	ΔH^0 (kJ/mol)	ΔS^0 (J/mol/K)
298	1.349480388	-3.27	12.54	53.04
308	1.430746124	-3.80		
318	1.643235292	-4.33		
328	1.79473124	-4.86		

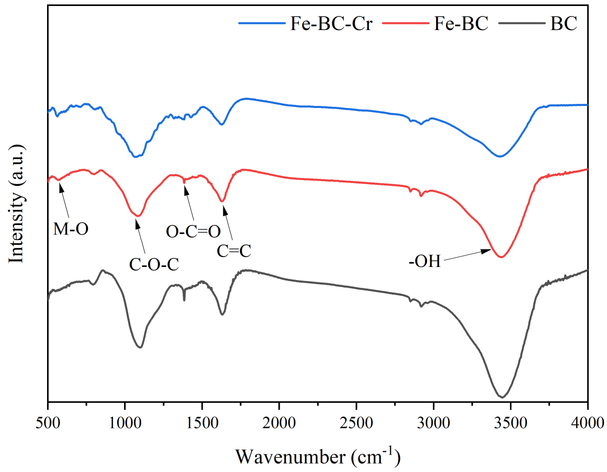

Figure S1. The FTIR spectrum of BC, Fe-BC and Fe-BC-Cr.

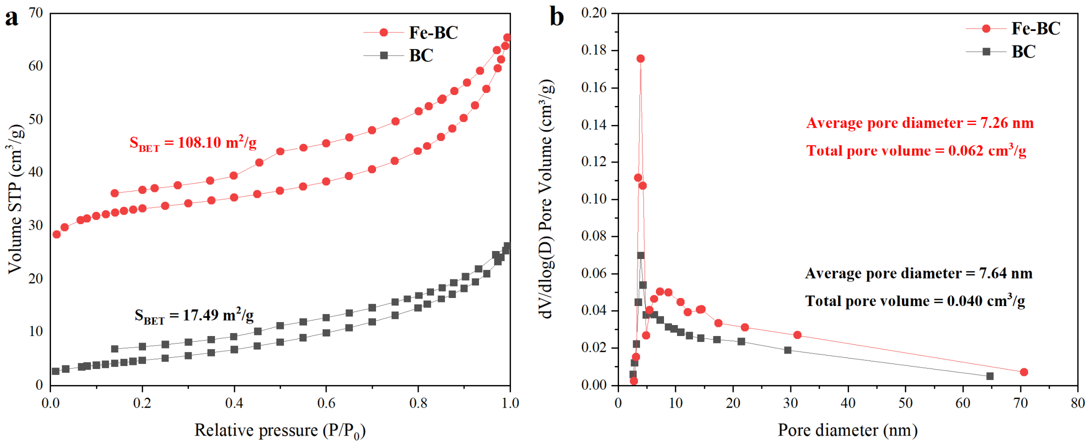

Figure S2. N_2 adsorption-desorption isotherm (a) and pore size distribution (b) for BC and Fe-BC.

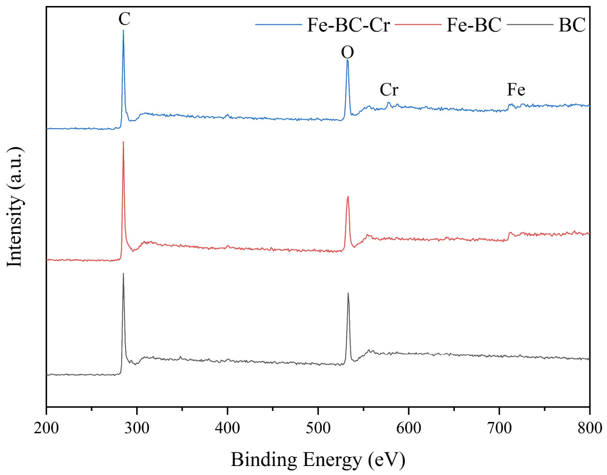

Figure S3. The survey XPS spectra of BC, Fe-BC and Fe-BC-Cr.

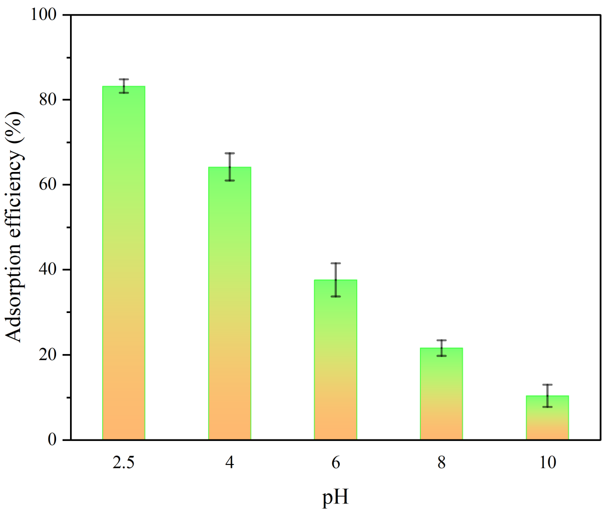

Figure S4. The effect of solution pH on the adsorption efficiency.

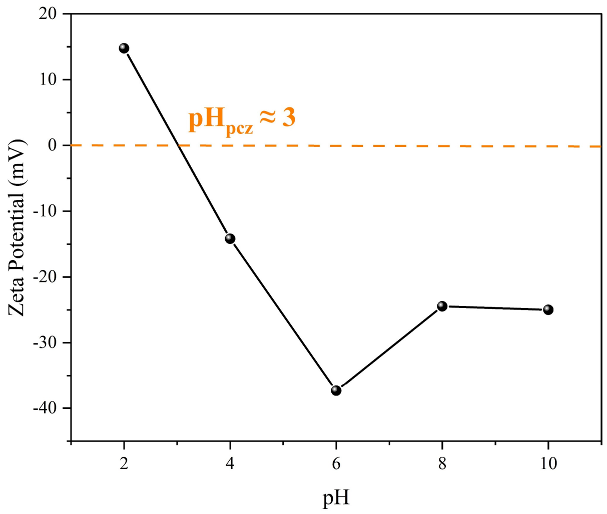

Figure S5. The Zeta potential of Fe-BC.

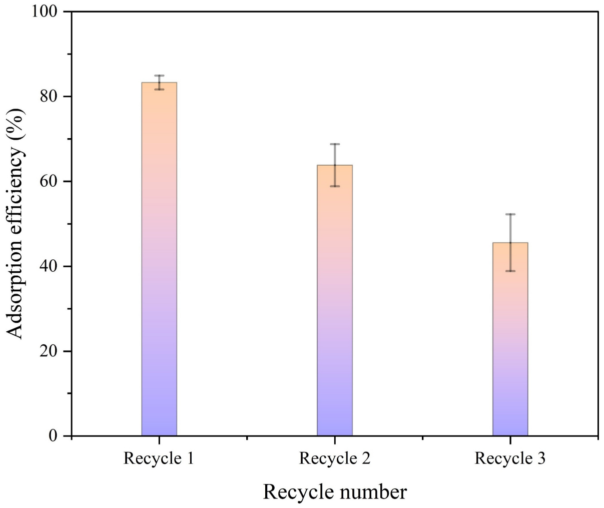

Figure S6. Regeneration performance of Fe-BC.
